# Imaging of underground karst water channels using an improved multichannel transient Rayleigh wave detecting method

**DOI:** 10.1371/journal.pone.0199030

**Published:** 2018-06-08

**Authors:** Xuhui Zheng, Lei Liu, Jinzhong Sun, Gao Li, Fubiao Zhou, Jiemin Xu

**Affiliations:** 1 School of Engineering and Technology, China University of Geosciences (Beijing), Beijing, P. R. China; 2 Institute of Disaster Prevention, East Yanjiao, Beijing, P. R. China; 3 China Non-ferrous Metals Resource Geological Survey, Beijing, P. R. China; China University of Mining and Technology, CHINA

## Abstract

Geological and hydrogeological conditions in karst areas are complicated from the viewpoint of engineering. The construction of underground structures in these areas is often disturbed by the gushing of karst water, which may delay the construction schedule, result in economic losses, and even cause heavy casualties. In this paper, an innovative method of multichannel transient Rayleigh wave detecting is proposed by introducing the concept of arrival time difference phase between channels (TDP). Overcoming the restriction of the space-sampling law, the proposed method can extract the phase velocities of different frequency components from only two channels of transient Rayleigh wave recorded on two adjacent detecting points. This feature greatly improves the work efficiency and lateral resolution of transient Rayleigh wave detecting. The improved multichannel transient Rayleigh wave detecting method is applied to the detection of karst caves and fractures in rock mass of the foundation pit of Yan’an Road Station of Guiyang Metro. The imaging of the detecting results clearly reveals the distribution of karst water inflow channels, which provided significant guidance for water plugging and enabled good control over karst water gushing in the foundation pit.

## Introduction

Karst geomorphology is widely distributed in southwestern China [1]. Underground constructions in karst areas are often threatened by karst water bursting and water gushing. Therefore, preliminary detecting and imaging of underground karst water channels in construction areas are of great significance.

The Rayleigh wave detecting method is an effective means for the imaging of underground medium space. The British scholar Rayleigh first discovered the existence of Rayleigh wave and revealed the propagation characteristics of this kind of wave in an elastic half-space medium in 1885 [2]. Subsequently, studies on engineering exploration methods of surface waves began. In the 1950s, Thomson [3] and Haskell [4]found the dispersion of Rayleigh waves propagating in layered media, following more studies focused on the dispersion characteristics of Rayleigh waves relative to layered media [5–7] and their application in the study on the structure of the Earth [8, 9]. In 1973, Chang and Ballard started using transient Rayleigh waves to study geological problems [10]. In 1982, the Japanese VIC Corporation developed a GR-80 type automatic survey machine for engineering geological investigation with steady-state Rayleigh waves [11]; however, its development was limited by its high price and low work efficiency. In 1983, Stoke and Nazarian et al. proposed a spectral analysis method of surface waves (SASW) [12]. Subsequently, the SASW method was improved and applied to a variety of engineering practices. In 1999, Xia et al. proposed a method of estimating the near surface shear-wave velocities by the inversion of Rayleigh waves, which greatly improved the transient Rayleigh wave detecting method [13]. In the early 21st century, in order to detect near surface discontinuities in materials, Zerwer et al. simulated Rayleigh wave propagation in the media with near surface discontinuities by finite element modeling [14]. Since then, the Rayleigh wave approach has been used for nondestructive detection in many fields [15–17].

In recent years, the Rayleigh wave detecting method has been widely used in geological and geophysical fields including explorations of water, oil, gas, and other subsurface geological detections [18]. Wills [19] and Cox [20] carried out engineering site investigation and assessment according to the dispersion characteristics of Rayleigh wave. Sun et al. studied the propagation characteristics of Rayleigh waves and successfully applied the Rayleigh wave detecting method to engineering problems of different scales [21]. Zerwer et al. introduced a nonlinear Rayleigh surface wave method to detect breaking cracks and micro-cracking in concrete members [22, 23]. Olivera et al. characterized stress corrosion cracking in carbon steel using P and Rayleigh waves [24]. Their conclusions showed that the approach of Rayleigh wave detecting is reliable for assessing deformations in different materials. Lin and Ashlock presented a study on the application of multichannel simulation with one-receiver (MSOR) surface-wave testing for geophysical profiling of soil sites, which showed another approach of Rayleigh wave detecting method [25].

In the field of geotechnical engineering and engineering geological reconnaissance, transient Rayleigh wave detection method is also widely used [17, 21, 26, 27], and it has been incorporated into the code for the measurement method of dynamic properties of subsoil [28] and other national standards in China.

As mentioned above, the Rayleigh wave detecting method has been developed and applied adequately. However, confined by the conventional sampling theorem, which states that the spacing of two adjacent detection points must be smaller than a certain portion of a wavelength [29], the existing multichannel transient Rayleigh wave detecting technique uses an array of multichannel geophones along a detecting line for picking up transient Rayleigh waves. Determined by the minimum channel spacing of the array, the phase velocity *V*_R_(*f*_max_) of the highest-frequency *f*_max_ fluctuant component can be extracted; meanwhile, determined by the maximum channel spacing of the array, the phase velocity *V*_R_(*f*_min_) of the lowest-frequency *f*_min_ can be extracted. Between the minimum and the maximum, a series of different channel spacing within the array can be formed, and therefore a series of phase velocities *V*_R_(*f*) of different frequencies *f* can also be extracted. As a result, only one curve of phase velocity-depth (*V*_R_ − *Z*) can be yielded from this array of multichannel geophones, which may be regarded as some kind of average distribution of phase velocities on depths within the scope covered by the geophone array. Obviously, this approach cannot form an imaging profile of Rayleigh wave velocities (*V*_R_(*X*, *Z*), where 0 ≤ *X* ≤ *L*, *L* is the scope covered by the array along measuring line *X*; 0 ≤ *Z* ≤ *Z*_max_, *Z*_max_ is the maximum depth of the depths *Z* detected by the array), which can reflect the changes over the range of the geophone array and within the scope of a certain depth. This deficiency results in low efficiency of in situ operation and a poor resolution of lateral variation in the properties of rock and soil masses.

In order to overcome the above-mentioned shortcomings of the existing transient Rayleigh wave detecting method, in this paper, the concept of arrival time difference phase between channels (TDP for short) is defined and the significance of the concept for extracting phase velocities of Rayleigh waves is clarified. Furthermore, a new detecting method considering the TDP of multichannel transient Rayleigh waves is proposed. The proposed method considers the propagation characteristics of Rayleigh waves and makes full use of the recorded information of every geophone, thereby greatly improving the lateral resolution and work efficiency of transient Rayleigh wave detecting. The efficiency and accuracy of this method were validated by applying it to the detection of karst water inflow channels in Guiyang Metro construction.

## Background: Phase velocity analysis of Rayleigh wave and arrival time difference phase between channels

### Phase velocity analysis of steady Rayleigh wave

In the light of wave stimulation approaches, there are two types of Rayleigh wave detecting, i.e., steady stimulation detecting and transient stimulation detecting, and phase-velocity extraction from steady stimulation Rayleigh waves is fundamental to the understanding of phase-velocity extraction from transient stimulation Rayleigh waves. During steady Rayleigh wave detecting, a series of single-frequency Rayleigh waves are sequentially excited by a series of harmonic vibrations of different frequencies generated by an apparatus at the stimulating point (source), and the phase velocity *V*_R_(*f*) of frequency *f* between two detecting points can be determined by the arrival time difference Δ*t* of the same phase points (time-difference method) or by the phase difference ΔΦ of the same moment (phase-difference method) between two waveforms on two detecting points (the spacing of the two detecting points is Δ*x*).

**Fig 1:**
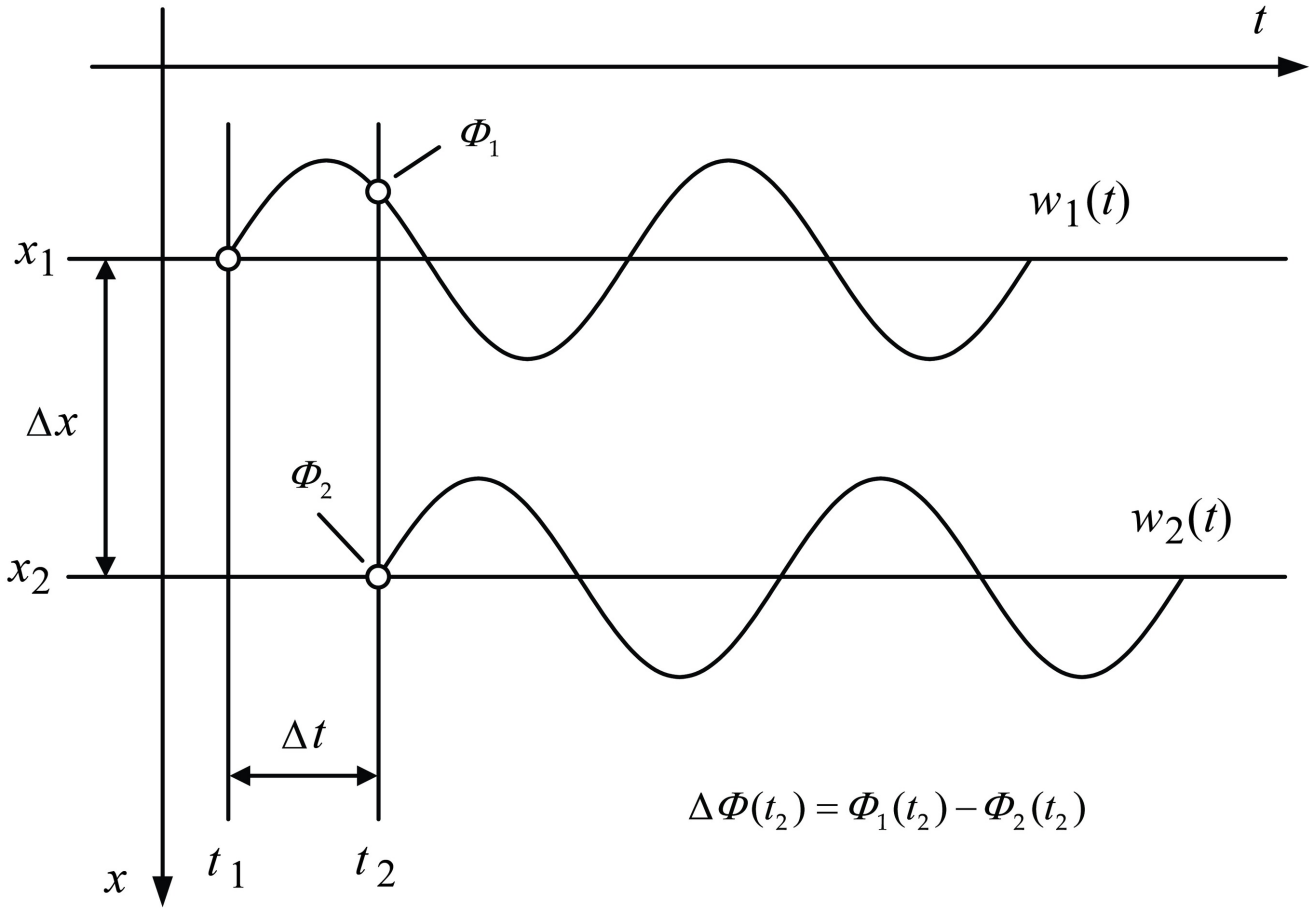
Sketch of the phase-velocity extraction from steady Rayleigh waves.

As shown in Fig 1, the axis *x* is the distance away from the simulating point (*x* = 0) along the detecting line; the axis *t* indicates the vibrating duration of the detecting points caused by wave propagation, and *t* = 0 corresponds to the beginning of the stimulating action. Assume that the vertical displacement *w* of particle vibration induced by Rayleigh wave propagation on any detecting point *x* of the detecting line can be expressed as a harmonic function as given by Eq (1):

$$\begin{array}{c} w=A\text{cos}\omega (t-\frac{x}{V_{\text{R}}}), \end{array}$$ (1)

where the amplitude *A* is a constant, *x* is the wave propagation distance, *ω* = 2*πf* is the circular frequency, *f* is the frequency, *t* is the wave propagation time, and *V*_R_ is the phase velocity of the corresponding frequency *f*.

The phase function of particle vibrating displacement is defined by Eq (2):

$$\begin{array}{c} \Phi \left(x,t\right)=\omega \left(t-\frac{x}{V_{\text{R}}}\right). \end{array}$$ (2)

On the same detecting line, a harmonic Rayleigh wave arrives at the two detecting points *x*_1_ and *x*_2_ (*x*_1_ < *x*_2_) successively, and the corresponding phase functions are Φ_1_ and Φ_2_, respectively:

$$\begin{array}{c} \Phi_{1}=\omega \left(t_{1}-\frac{x_{1}}{V_{\text{R}}}\right),\Phi_{2}=\omega \left(t_{2}-\frac{x_{2}}{V_{\text{R}}}\right). \end{array}$$ (3)

Let Δ*t* = *t*_2_ − *t*_1_ and Δ*x* = *x*_2_ − *x*_1_, and the phase difference ΔΦ can be expressed by Eq (4):

$$\begin{array}{c} \Delta \Phi =\Phi_{1}-\Phi_{2}=\omega \left(\frac{\Delta x}{V_{\text{R}}}-\Delta t\right). \end{array}$$ (4)

For equal phase points of the vibrations at the two detecting points, ΔΦ = Φ_1_ − Φ_2_ = 0, and Δ*t* = *t*_2_ − *t*_1_≠0. The phase velocity formula can be deduced from Eq (4),

$$\begin{array}{c} V_{\text{R}}\left(f\right)=\frac{\Delta x}{\Delta t}. \end{array}$$ (5)

Eq (5) is the calculation formula for extracting phase velocity of Rayleigh wave by the time-difference method.

On the other hand, to the same moment, *t*_1_ = *t*_2_ (that is, Δ*t* = 0), and Φ_1_ ≠ Φ_2_, ΔΦ = Φ_1_ − Φ_2_ ≠ 0. Thus, another phase velocity formula can also be deduced from Eq (4), as shown by Eq (6):

$$\begin{array}{c} V_{\text{R}}\left(f\right)=\frac{2\pi f\Delta x}{\Delta \Phi }. \end{array}$$ (6)

Eq (6) is the calculation formula for extracting the phase velocity of Rayleigh wave by the phase-difference method.

By changing stimulating frequency *f* successively, a series of phase velocities corresponding to various frequencies can be obtained with Eqs (5) or (6).

### Phase velocity analysis of transient Rayleigh wave

Unlike steady Rayleigh waves, transient Rayleigh waves, stimulated by a strong impact (a transient pulse force), are complex waves of many harmonic components. Therefore, two channels of transient Rayleigh waveforms picked up on a pair of different detecting points contain information about the propagation of every harmonic component. This means that the phase velocities of different frequencies can be extracted from these two transient Rayleigh waveforms stimulated by only one impact. In contrast to steady Rayleigh waves, many times of harmonic vibrating stimulations are required to extract the same phase velocities of different frequencies with those from the two transient Rayleigh waveforms. Furthermore, just because transient Rayleigh waves are complex waves, more analyses are required to extract every harmonic component from the complex wave, and the extraction of phase velocities from transient Rayleigh wave will not be as simple as that from steady Rayleigh wave.

In order to extract phase velocities of different frequencies from two channel waveforms of transient Rayleigh waves picked up on a pair of different detecting points, three steps of analysis work are needed: firstly, confirm the section of transient Rayleigh wave in each waveform based on wave phase analysis; secondly, decompose the section of transient Rayleigh wave into a series of harmonic components with different frequencies by means of Fourier transform; and lastly, calculate the phase difference and phase velocity of each harmonic component by phase difference method.

**Fig 2:**
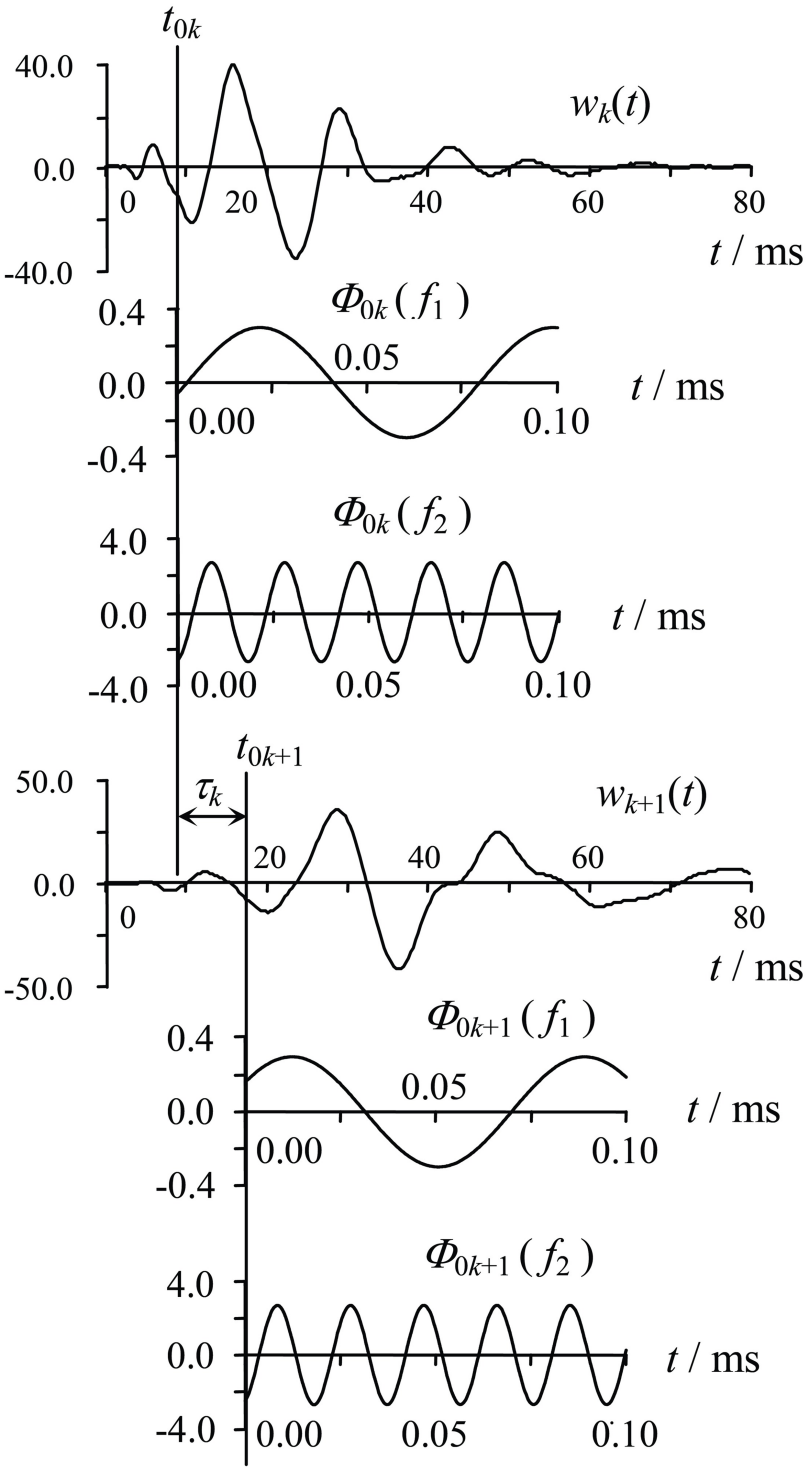
Sketch illustrating the calculation of the time difference phase of a transient Rayleigh wave.

As shown in Fig 2, to confirm the section of the transient Rayleigh wave in the waveform picked up at each detecting point, wave phase analysis based on the kinematic and dynamic features of transient Rayleigh wave propagation is helpful [30]. Through careful wave phase analysis, two time parameters of the Rayleigh wave section can be obtained: the arrival time *t*_0*k*_ (the starting moment of the section) and the duration *T*_R*k*_ (the lasting time of the section, or the length of the time window of Rayleigh wave) of Rayleigh waves in the waveform picked up on the detecting point. A filtering method is used to eliminate the interference of other wave phases such as P and S waves in the recorded waveforms. The confirmed section of transient Rayleigh waves in a waveform picked up on a detecting point is expressed as Eq (7):

$$\begin{array}{c} w_{k}\left(t\right),\left(k=1,2,\ldots ,n;t\in \left[t_{0k},t_{\text{R}k}\right]\right), \end{array}$$ (7)

where *k* is the serial number of a detecting point on a detection line; *n* is the total number of detecting points of the detecting line; *t* is the fluctuation time; [*t*_0*k*_, *t*_R*k*_] is the sampling time window of the recorded waveform of the *k*^th^ detecting point; *t*_0*k*_ is the starting time of the sampling time window; *t*_R*k*_ = *t*_0*k*_ + *T*_R*k*_ is the end of the sampling time window; and *T*_R*k*_ = *t*_R*k*_ − *t*_0*k*_ is the duration of the transient Rayleigh wave at the *k*^th^ detecting point.

After confirmation through the wave phase analysis and filter process, the section of transient Rayleigh waves in the waveform shown in Eq (7) is ready to be decomposed into a series of harmonic vibration processes of different single frequencies by means of Fourier transform technique as shown by Eq (8):

$$\begin{array}{c} w_{kj}\left(t\right)=A_{k}\cdot \text{cos}\left(\omega_{j}\cdot t+\Phi_{0k}\right). \end{array}$$ (8)

In Eq 8, *w*_*kj*_(*t*) is a harmonic vibration displacement process of frequency *f*_*j*_ originating from the transient Rayleigh wave of channel *k* (recorded on detecting point *k*), and *ω*_*j*_ = 2*πf*_*j*_ is the circular frequency of the harmonic vibration; *A*_*k*_ = *A*_*k*_(*f*_*j*_) is the amplitude of the displacement process; and Φ_0*k*_ = Φ_0*k*_(*f*_*j*_) is the initial phase of the section, the phase at the start of the sampling window.

According to the harmonic vibration processes shown in Eq (8), last step of phase velocity extraction from the transient Rayleigh waves of two channels can be implemented. Because it is difficult to determine the same phase points for different frequency components from two channel waveforms of transient Rayleigh waves, the phase-difference method is adopted to calculate the phase velocities. Applied in different frequency components of transient Rayleigh waves shown in Eq (8), the calculation formula for phase velocity of Rayleigh wave Eq (6) should be in the form of Eq (9) as follows:

$$\begin{array}{c} V_{\text{R}k}\left(f_{j}\right)=\frac{2\pi f_{j}\Delta x_{k}}{\Delta \Phi_{k}\left(f_{j}\right)}, \end{array}$$ (9)

where Δ*x*_*k*_ = *x*_*k*+1_ − *x*_*k*_ is the distance between detecting points *k* and *k* + 1; and ΔΦ_*k*_(*f*_*j*_) = Φ_*k*_(*f*_*j*_) − Φ_*k*+1_(*f*_*j*_) is the phase difference of harmonic components with frequency *f*_*j*_ of the transient Rayleigh waves originated from detecting points *k* and *k* + 1.

### Arrival time difference phase between channels

As mentioned above, similar to Eq (4), the phase difference ΔΦ_*k*_(*f*_*j*_) of harmonic components with frequency *f*_*j*_ should be expressed by Eq (10):

$$\begin{array}{c} \Delta \Phi_{k}\left(f_{j}\right)=\Phi_{k}\left(f_{j}\right)-\Phi_{k+1}\left(f_{j}\right). \end{array}$$ (10)

According to Eqs (8) and (10) should be further written as:

$$\begin{array}{c} \Delta \Phi_{k}\left(f_{j}\right)=\Delta \Phi_{0k}\left(f_{j}\right)+2\pi f_{j}\tau_{k}. \end{array}$$ (11)

In the equation,

$$\begin{array}{c} \Delta \Phi_{0k}\left(f_{j}\right)=\Phi_{0k}\left(f_{j}\right)-\Phi_{0k+1}\left(f_{j}\right), \end{array}$$ (12)

$$\begin{array}{c} \tau_{k}=t_{k+1}-t_{k}=t_{0k+1}-t_{0k}. \end{array}$$ (13)

*τ*_*k*_ may be treated as the arrival time difference (travel time) of the transient Rayleigh wave propagating between detecting points *k* and *k* + 1; 2*πf*_*j*_*τ*_*k*_ is the phase difference related to the arrival time difference *τ*_*k*_. Here, we define the term 2*πf*_*j*_*τ*_*k*_ as the arrival time difference phase between channels, abbreviated as TDP.

In the phase velocity extraction by existing multichannel transient Rayleigh wave detecting technique, TDP is ignored, and, to eliminate the influence owing to the absence of TDP, the distance between two geophones is limited to a length less than a wavelength [29].

## Method: Multichannel transient Rayleigh wave detecting method considering TDP

With only two channel waveforms recorded at two detecting points, a curve of the Rayleigh wave phase velocity vs. effective detecting depth (*V*_R_ − *Z*) can be extracted after introducing TDP; this approach greatly improves the efficiency of in situ operation and the lateral resolution of multichannel transient Rayleigh wave detecting. First, from a detecting line of multichannel detectors with certain channel spacing, more than one curve of phase velocity vs. effective detection depth between pairs of adjacent detecting points can be acquired. Second, by suitable detecting network coverage of multiple detecting lines with certain spacing between the detecting lines, highly efficient and continuous imaging of the foundation soil mass can be achieved. This novel method of multichannel transient Rayleigh wave detecting may be named TDP method. The implementation process of TDP method is described in detail below.

### Determination of working frequency band

The working frequency band of transient Rayleigh wave detecting depends on the requirements of detecting depth and the shallow layer resolution. The detecting depth determines the low limit of the working frequency, and the shallow layer resolution determines the high limit.

According to wave theory, the relationship among the Rayleigh wavelength λ_R_, phase velocity *V*_R_, and frequency *f* is given as follows:

$$\begin{array}{c} f=V_{\text{R}}/\lambda_{\text{R}}. \end{array}$$ (14)

In addition, the relationship between the effective detecting depth *Z* and the wavelength λ_R_ is:

$$\begin{array}{c} Z=\beta \cdot \lambda_{\text{R}}, \end{array}$$ (15)

where *β* is a coefficient related to the Poisson’s ratio of soil or rock mass in which Rayleigh wave is propagating, as indicated in Table 1.

**Table 1:** Effective detection depth of Rayleigh waves in different media [31].

Poisson’s ratio *ν*	0.10	0.15	0.20	0.25	0.30	0.35	0.40	0.45	0.48
*β* = *H*/λ_*R*_	0.55	0.575	0.625	0.65	0.7	0.75	0.79	0.84	0.875

From Eqs (14) and (15), the following result can be acquired:

$$\begin{array}{c} f=\beta \cdot V_{\text{R}}/Z. \end{array}$$ (16)

Set the required maximum detecting depth and the shallowest resolution depth of soil or rock mass as *Z*_max_ and *Z*_min_ respectively, and substitute the depth *Z* in Eq (16) with *Z*_max_ and *Z*_min_ separately. Consequently, the lowest frequency *f*_D_ and highest frequency *f*_U_ can be determined as follows:

$$\begin{array}{c} f_{\text{D}}=\beta \cdot V_{\text{R}}/Z_{\text{max}},\;f_{\text{U}}=\beta \cdot V_{\text{R}}/Z_{\text{min}}. \end{array}$$ (17)

### Layout of the observation system

The layout of the observation system for transient Rayleigh wave detecting is determined by three parameters: the channel spacing between detecting points, the lengths of detecting lines, and the intervals between detecting lines. With the introduction of TDP, the limitation on the channel spacing (the distance between two adjacent detecting points) may be eliminated. Therefore, recognizing lateral changes of soil or rock mass properties becomes the main factor controlling the spacing of detecting points and lines. As long as the properties of soil or rock mass between two adjacent detecting points are unchanged, it is advisable to increase the channel spacing as large as possible; even variations of channel spacing is also acceptable under some conditions. Using this approach, the detecting workload can be reduced reasonably, and a relatively high accuracy of measuring the arrival time difference of Rayleigh waves between channels is ensured. For the convenience of three-dimensional imaging of Rayleigh wave phase velocities of soil or rock mass, it is suggested that the extending direction of detecting lines be parallel to the long axis of the site to be detected, and the spacing between detecting lines be the same as or similar to the spacing of detecting points.

### Stimulation and observation of transient Rayleigh waves

**Fig 3:**
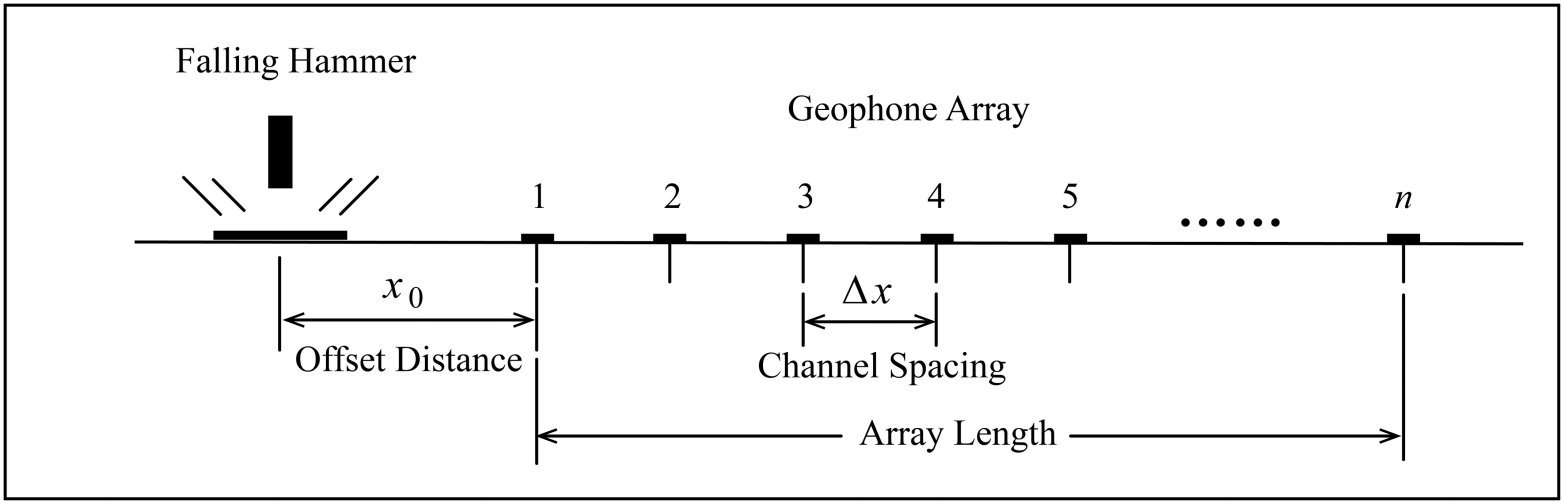
Sketch of the transient Rayleigh wave stimulation and observation system.

As shown in Fig 3, a transient Rayleigh wave is stimulated by an impulse force caused by a heavy hammer falling onto the stimulating point, and an array of multiple channel geophones with channel spacing Δ*x* and offset distance *x*_0_ is laid along a detecting line. The position of the stimulating point (controlled by offset distance) and hammer weight are parameters related to the stimulation of transient Rayleigh waves, and channel spacing, number and natural frequency of geophones are parameters to lay the observation system of transient Rayleigh wave.

**Fig 4:**
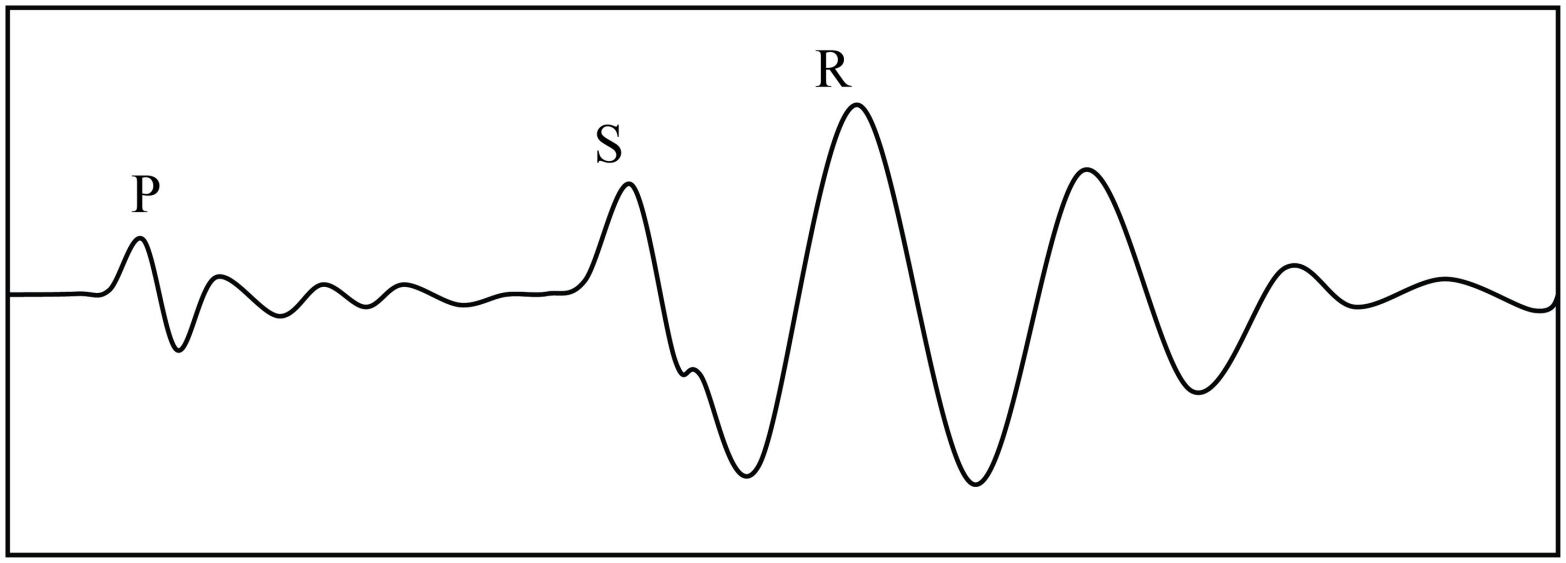
Time-domain waveform of a single channel record.

The stimulating point should be along the extension direction of a detecting line, and offset distance (the distance between the stimulating point and the nearest detecting point) should be sufficiently long such that there is enough time lag between Rayleigh wave and its preceding wave phases such as S wave and P wave in the time-domain waveform recorded on any detecting point (as shown in Fig 4). It will be helpful to extract the Rayleigh wave section from the waveform. Chen et al. found that the relationship between the offset distance *L* and the largest wavelength λ_max_ can be expressed as *L* = *αλ*_max_, where *α* is in the range of 0.3^~^2.0 [29].

The working frequency band of transient Rayleigh wave detecting can be adjusted by the weight of falling hammer and the natural frequency of detecting geophones. The hammer weight determines the predominant frequency range (the frequency band in which a large portion of the wave energy lies) of the transient Rayleigh wave: the heavier the hammer is, the lower predominant frequency band will be stimulated. The natural frequency of geophones determines the frequency range of the Rayleigh waves picked up by the geophones, because the wave vibration recorded by a geophone is the response of the geophone to the fluctuation of the detecting point and the response frequency is always close to the natural frequency of the geophones. In the practice of transient Rayleigh wave detecting, proper matching between the weight of falling hammer and the natural frequency of geophones should be determined by field tests to satisfy the requirement of the working frequency band.

In practical operation, two stimulating points can be set separately on both ends of a detection line. Thus, the Rayleigh waves stimulated at these two stimulating points can complement each other, which will be helpful to improve the signal-to-noise ratio of detected waveforms.

High-quality recorded waveforms of transient Rayleigh waves should have the following features:

1) The signal-to-noise ratio is high, the Rayleigh wave signal is obvious, and undesired signals such as S wave and P wave are weak; 2) the initial motion of the Rayleigh wave is clear, and the event of multichannel Rayleigh waves is continuous; 3) the frequency components of the Rayleigh wave match the range of the objective detecting depth; and 4) the sampling repeatability of waveforms recorded at the same detecting point is good.

### Imaging of Rayleigh wave phase velocities

According to Eq (9) and the waveform data of Rayleigh waves obtained by an in situ observation system, phase velocities of the media between any two adjacent geophones along a detecting line can be extracted, from which the phase velocity vs. depth curves (*V*_R_ − *Z*) can be formed. In total, (*n* − 1) pieces of *V*_R_ − *Z* curves can be obtained from a detecting line with *n* detection points. Finally, colligating these *V*_R_ − *Z* curves, we can map the velocity distribution in rock and soil mass within a certain depth range under the detecting line (phase-velocity distribution of a vertical section).

By applying the same operation to all detecting lines of the observation system, the *V*_R_ − *Z* curves between all adjacent detection points of every detecting line can be obtained. With the collection of the phase-velocities above the same depth level from all detecting lines in the observation system, the distribution of the phase velocities above this depth level (phase-velocity distribution of a horizontal section) can be formed.

## Case: Detection of Karst water channels in rock mass under a foundation pit of Guiyang Metro

### Basic geological conditions of the work area

Yan’an road station (geographical coordinates 26.6°N, 106.7°E) on Line 2 of Guiyang Metro is a transfer station located in the central part of Guiyang city. According to geological data from field geological investigation, the strata of the station site area comprises Quaternary overburden layers and underlying Triassic bedrock. The Quaternary overburden layer mainly contains concrete, a stone layer, a gravel layer, miscellaneous soil, and red clay, the thickness of which has a certain difference because of the controlling undulation of the bedrock. The underlying Triassic bedrock is mainly dissolvable dolomite. Karst structures and fault breakage belts developing in the rock mass, filled with underground water, form the greatest obstacle and danger to the excavation of the station pit. Once water inrush occurs during excavation, not only the schedule will be delayed, but also the stability of the foundation pit be threatened, increasing the risk of further casualties. To determine the scale and location of the potential fault belts and karst caves in the rock mass under the foundation pit, the multichannel transient Rayleigh wave detecting method considering TDP is used to ascertain the distribution of water channels in the rock mass attributable to Karst action and fault breakage.

### On-site detection

**Fig 5:**
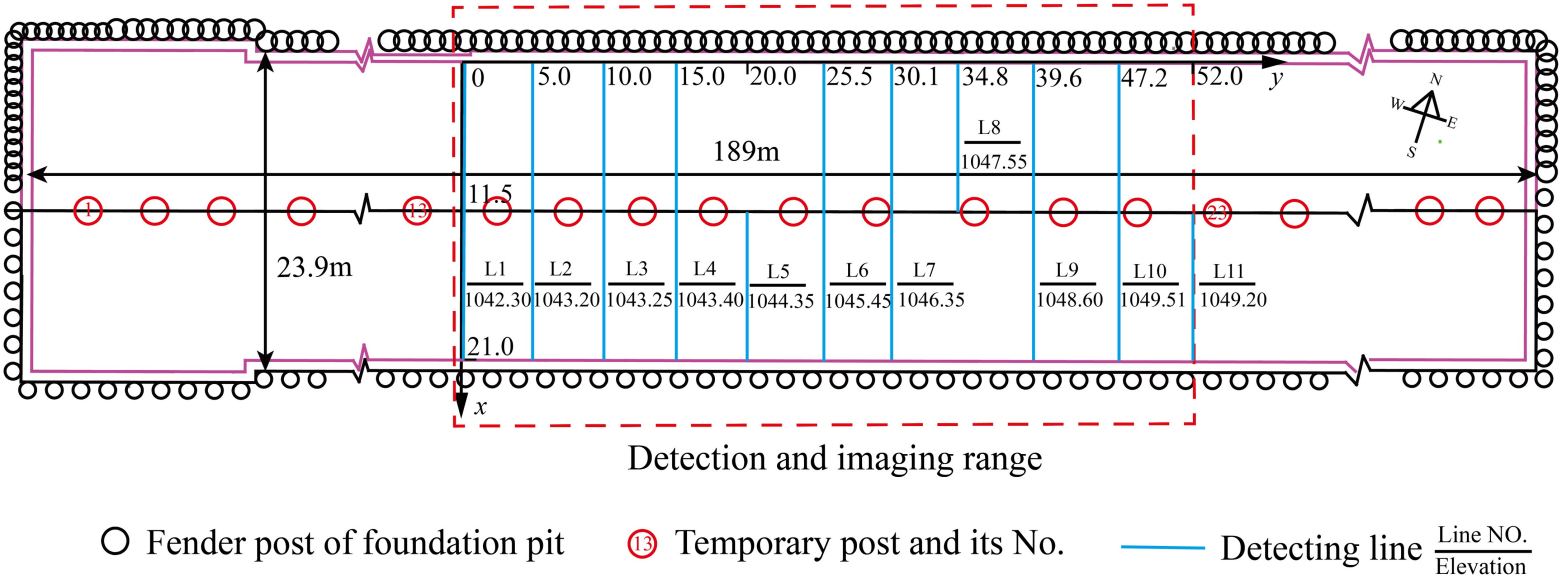
Sketch map of the observing system of Rayleigh wave detecting in the station pit.

Fig 5 shows the layout of Rayleigh wave detecting observing system between the temporary foundation fender posts No.13 and No.23 in the foundation pit of Yan’an road station. In this study, detecting line 2 was taken as an example to introduce the detecting method.

To generate lower frequency transient Rayleigh wave propagating in the rock mass, a layer of sand cushion (thicker than 10cm) and a thick rubber pad were placed on the ground at the stimulating point. Detecting waves were stimulated by a hammer of 128kg weight falling down to the ground from a height of 1.5m. SWG seismograph with 12 receiving channels and 12 geophones of model SG-5 with natural frequency 5Hz were used to pick up and record the waves propagating along the detecting line in the rock mass. The major specifications of the instrument are listed in Table 2. The practical detecting results show that the effective detecting depth exceeds 25m, which satisfies the requirement of the engineering project.

**Table 2:** Major specifications of SWG seismograph.

Parameters	Specifications
Analogue Channel	1^~^12 (Setting Arbitrary)
Gain	0^~^96dB
Frequency Band	0.5Hz^~^20kHz
Sampling	Each Spacing Point: 512^~^8192, Spacing Interval: 8*μ*s^~^15s
Delay times	0^~^10s
Storage	ROM: 72kB; System RAM: 192kB; Temporary Storage RAM: 1024kB
Geophone	Specifications Model: SG-5m, Dominant Frequency: 5Hz

**Fig 6:**
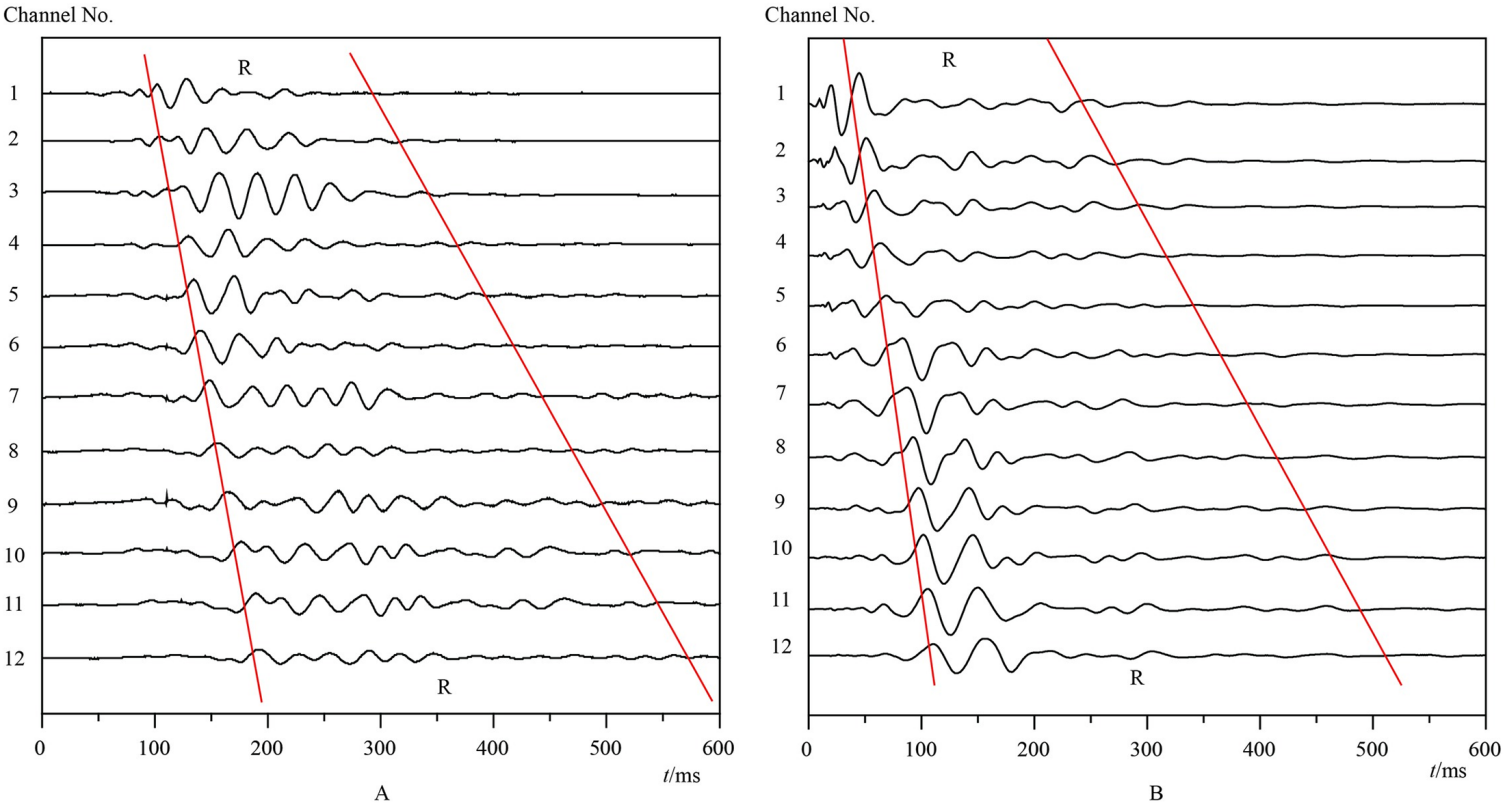
Recorded multichannel transient Rayleigh waveforms of detection line 2. A: Array 1. B: Array 2.

According to the configuration of the instrument and the detecting precision, the distance between adjacent measuring points was set as 1.0m. An array of 12 geophones with 1.0m channel spacing was designed, which has 11 intervals between adjacent geophones and can cover a scope of 11m along a detecting line. For example, two arrays were set end-to-end along detecting line 2 (L2 in Fig 5), covering a length of 21m. The waveform recorded on-site by these two arrays is shown in Fig 6.

### Imaging of underground karst water channels

**Fig 7:**
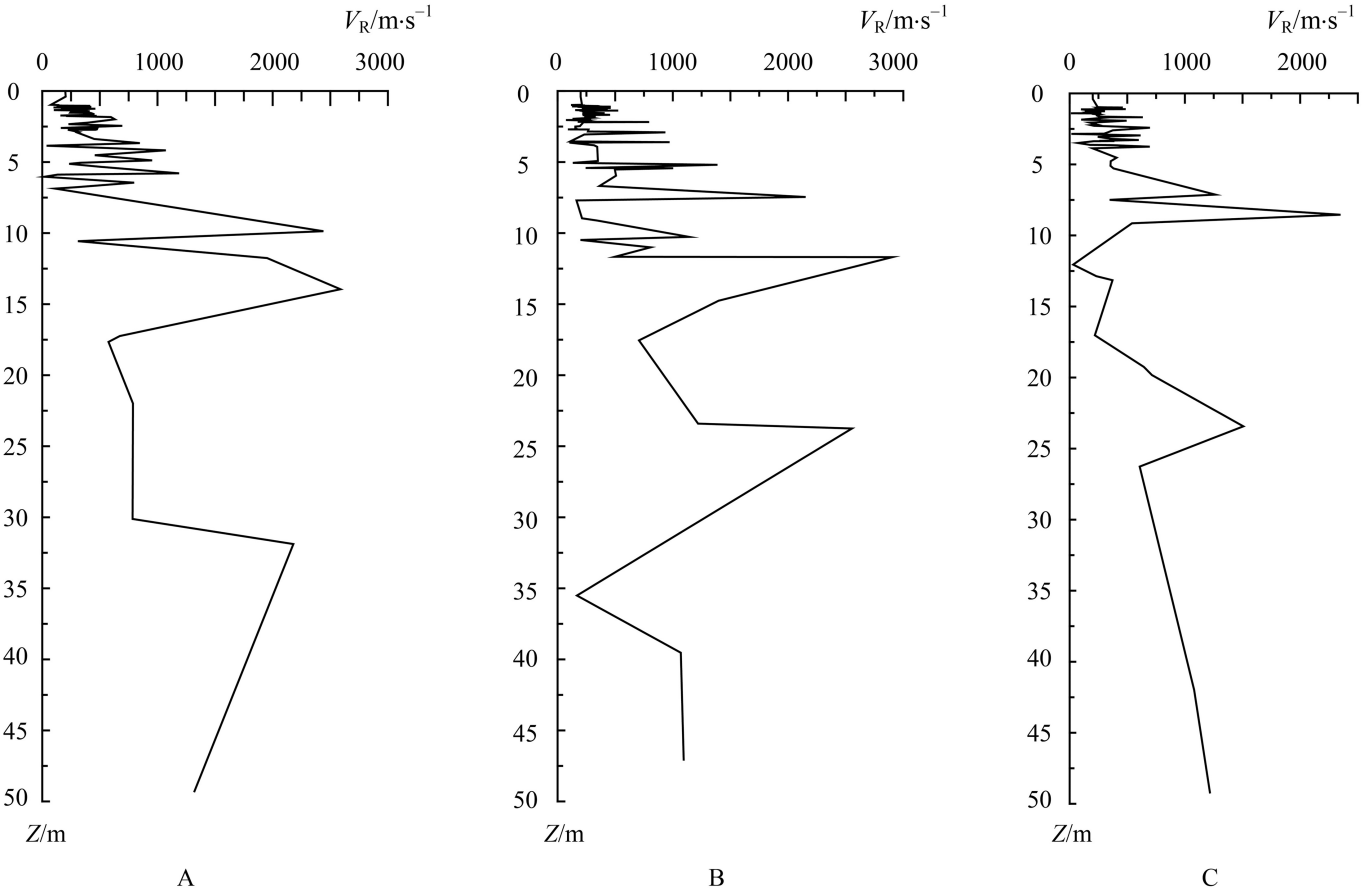
Curves of phase velocity vs. depth. A: detecting points 1-2. B: detecting points 3-4. C: detecting points 9-10.

**Fig 8:**
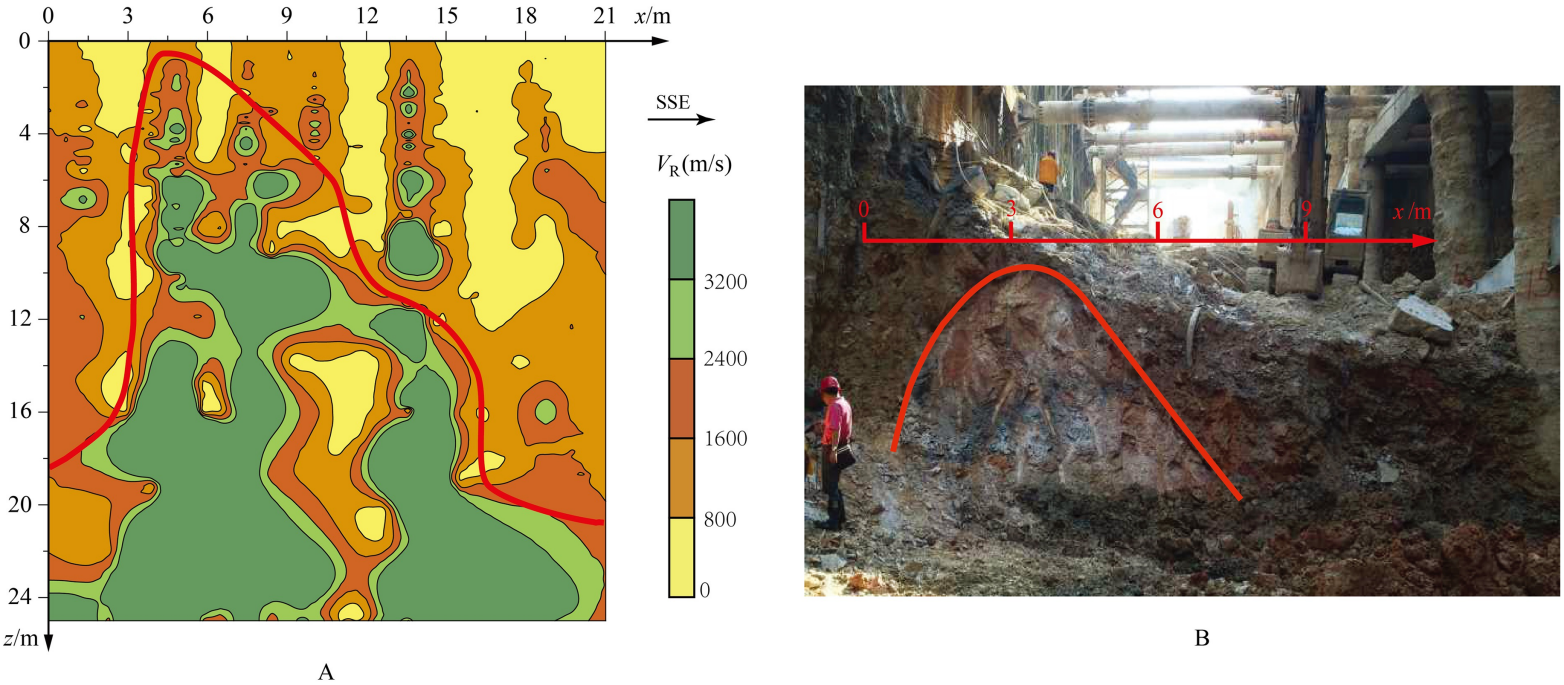
Comparison of vertical profile with the Rayleigh wave phase velocity and the geological section. A: vertical profile of the Rayleigh wave phase velocity. B: geological section of detection line 2.

With any two waveforms recorded on adjacent detecting points, the analysis method of multichannel transient Rayleigh wave detecting considering TDP can be used to generate the phase velocity-depth curve (*V*_R_ − *Z*), as shown in Fig 7. All the *V*_R_ − *Z* curves can be used to form an imaging profile along an entire detecting line. Fig 8 shows the imaging profile of detecting line 2. To confirm existing relationships between the velocity variation range of the rock and soil mass classification and the hardness of rock and soil mass, related content was referred from the Code for Seismic Design of Buildings [32]. According to the lithologic category and weathering degrees of rock mass, especially properties related to potential fault zones and karst zones, a new variation range of Rayleigh wave velocity related to different types of rock and soil mass was defined, as shown in Table 3.

**Table 3:** Rayleigh wave phase velocity classifications of rock and soil mass.

*V*_R_(m/s)	≤ 800	800^~^1600	1600^~^2400	2400^~^3200	≥ 3200
Rock and Soil Properties	Overlying soil mass, Fault fracture zones, Karst fissures, etc.	Strongly weathered rock mass	Medium weathered rock mass	Weakly weathered rock mass	Unweathered rock mass

As shown in Fig 8, the phase velocity profile of Rayleigh waves along the detecting line 2 is well consistent with the geological section. The phase-velocity range of 1600^~^2400m/s corresponds approximately to the geological section marked by the red curve. The phase velocity image profile of detecting line 2 shown in Fig 8A is well coincident with that in Fig 8B, both in shape and in position, with the geological section near detecting line 2 revealed by pit digging later.

**Fig 9:**
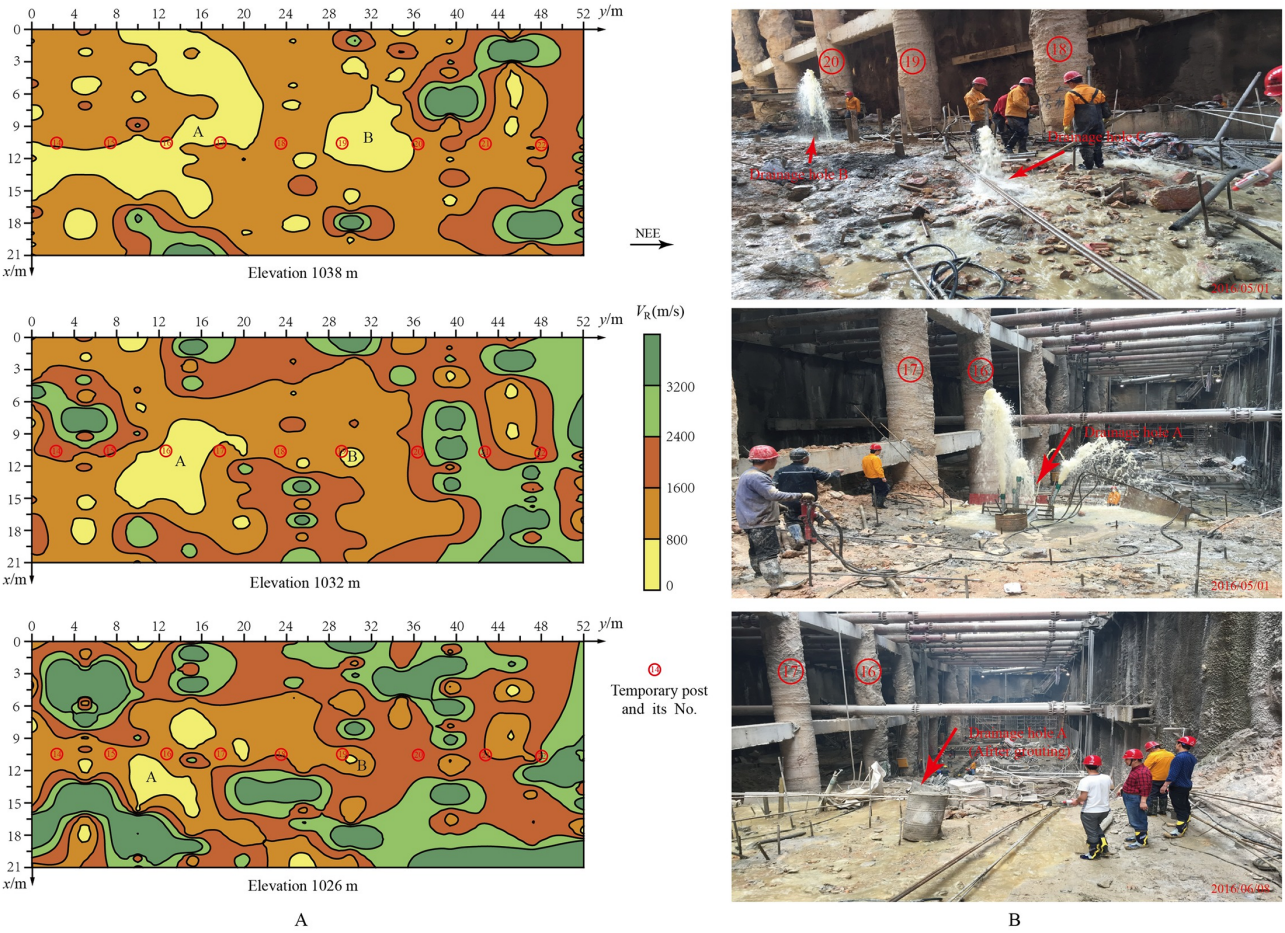
Comparison of horizontal imaging sections of Rayleigh wave phase velocities and the actual pictures on the bottom of the foundation pit. A: horizontal imaging section of phase velocities on different depth. B: photographs of water inflow in the foundation pit.

In the detection range, the phase-velocity information at a certain depth of all the detecting lines was extracted, and the phase-velocity distribution of the medium over that depth can be obtained. The phase-velocity distributions over elevations 1038m, 1032m and 1026m are shown in Fig 9A.

There are two major low-velocity zones in the phase-velocity imaging section at 1038m elevation, which indicates the distribution and range of karst zones on this section. Karst zone B is located between temporary post No.19 and No.20. Karst zone A spreads wider and mainly covers the range of temporary post No.13^~^No.17. With increasing depth, the distribution of karst zones is significantly reduced. At elevation 1032m, karst zone B has almost vanished, while karst zone A decreases to the range of temporary post No.16^~^No.17. At 1026m elevation, karst zone B has completely disappeared, and karst zone A also shrinks to a small area located on the north and south sides of temporary post No.16. These three horizontal phase velocity imaging sections clearly revealed the distribution of karst water channels in the rock mass.

According to the distribution of karst zones revealed by Fig 9A, three vertical drainage holes were set between temporary post No.16 and No.20 in later construction as shown in Fig 9B. Drainage hole A and C were set in karst zone A, and drainage hole B was set in karst zone B. From the two photographs taken on May 1st of 2016 in Fig 9B, it is clear that the water discharge amount at drainage hole A is significantly larger than that at drainage hole B and C, which also reflects the fact that karst zone A spread wider and deeper than karst zone B.

**Fig 10:**
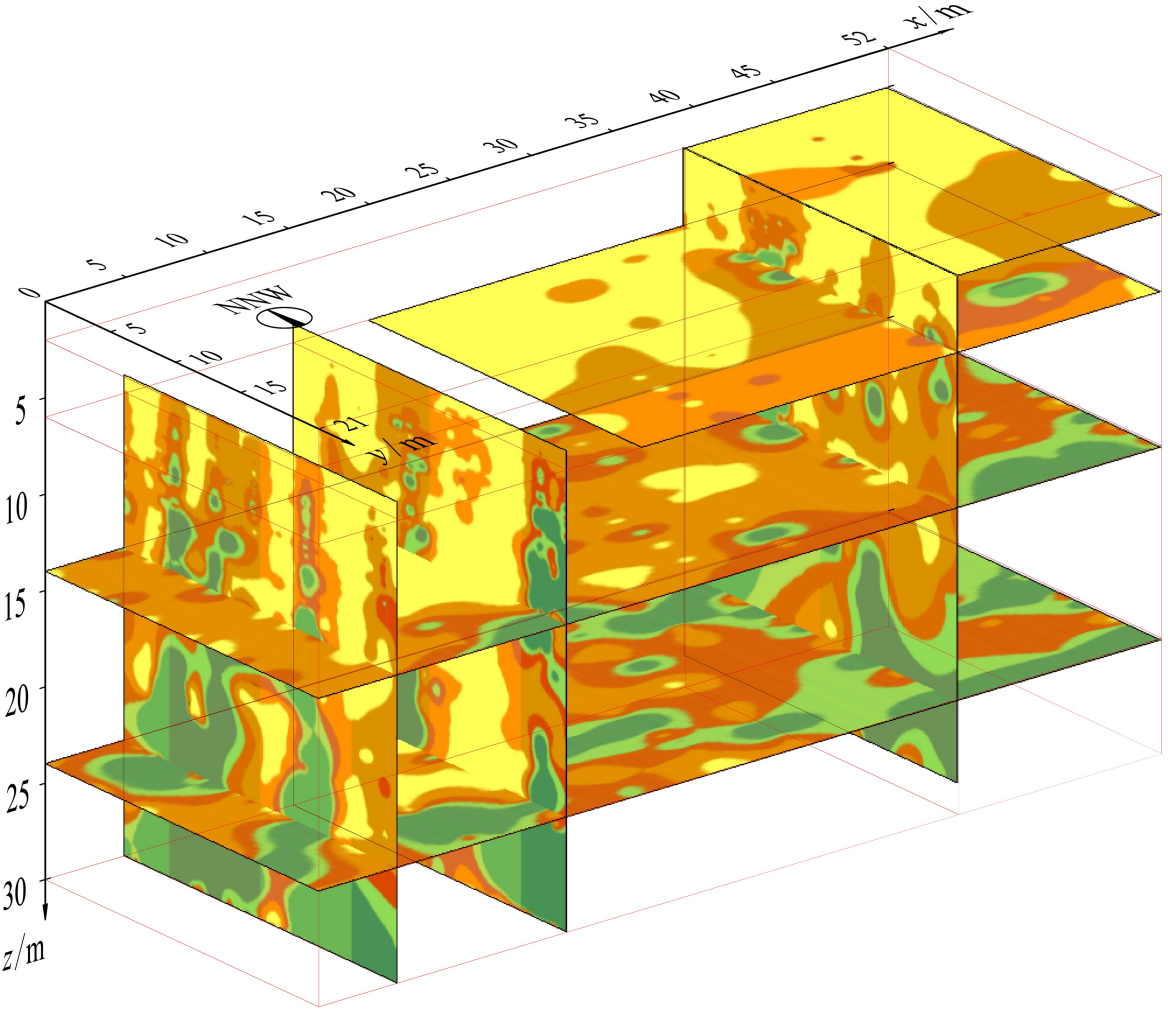
Quasi-3D imaging of the underground karst distribution in the foundation pit scope.

According to the on-site Rayleigh wave observation system, all the obtained vertical and horizontal profiles of the phase-velocity imaging were combined in a 3D coordinate system (Fig 10), thus establishing a quasi-3D phase velocity imaging of rock and soil masses (*V*_R_ − *x* − *y* − *z*). Based on the classifications in Table 3 and the geological conditions at the site, areas with phase velocities less than 800m/s are considered to be karst water-rich areas. The quasi-3D imaging intuitively reveals the scale and spatial distribution of the underground karst water channels, which provides strong technical support for the treatment of karst water gushing problems in the excavation process of the foundation pit. Targeted measures of drainage, filling, and grouting were taken to control waterborne diseases. As shown in the photograph taken on June 8th of 2016 in Fig 9B, the water gushing in the foundation pit has been successfully plugged.

## Discussion

### Limitations of the existing multichannel transient Rayleigh wave detecting method

**Fig 11:**
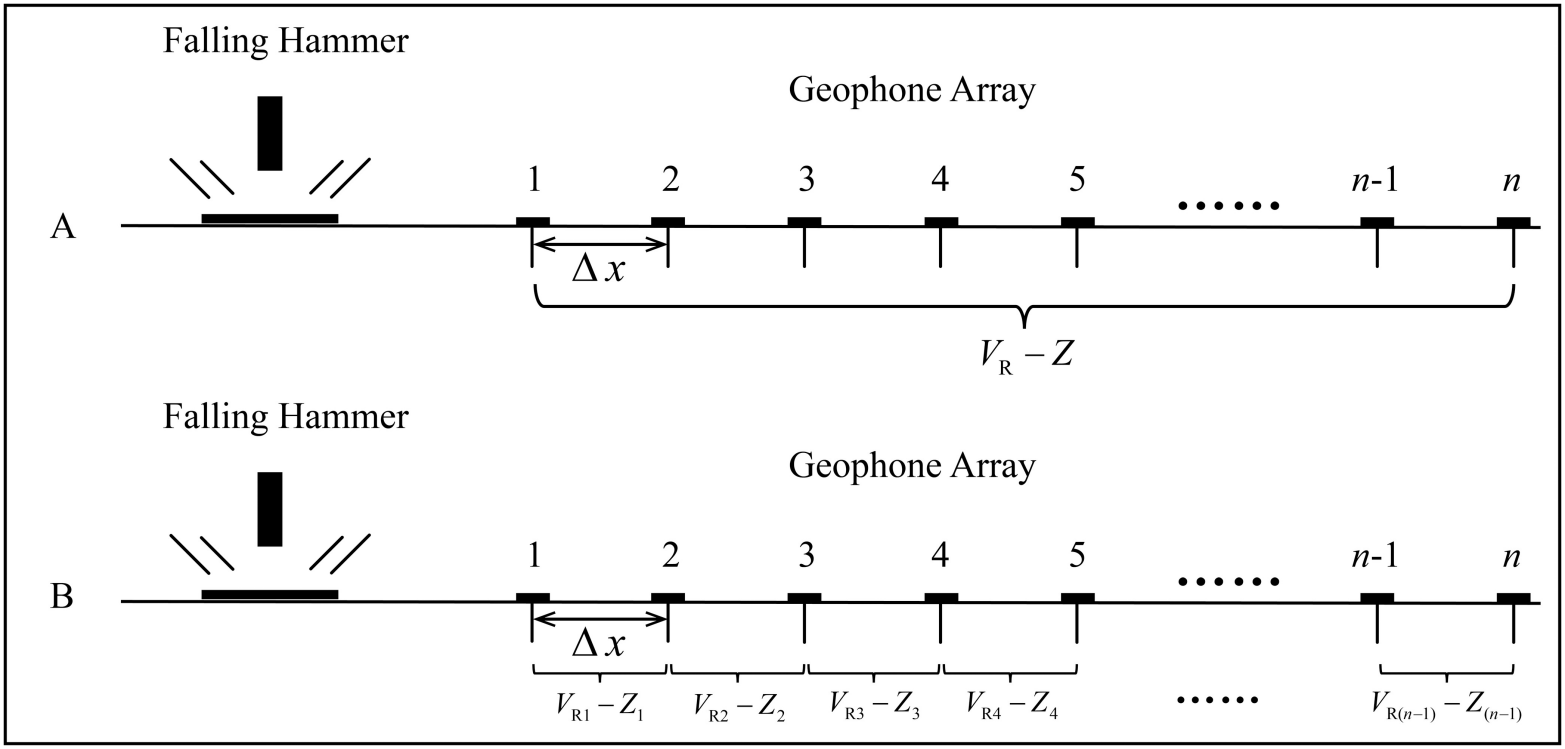
Sketch of the difference between the existing multichannel transient Rayleigh wave detecting method and the improved one. A: existing method. B: improved method.

1. In light of the concept of effective detecting depth of Rayleigh waves [29], in order to detect the properties of rock and soil masses over different depths of a site, a series of phase velocities corresponding to different frequency components (different wavelengths), i.e., dispersion curves of the Rayleigh waves, are required. To reveal the rock and soil masses at one point, the space between two detecting points (channel spacing) must be changed according to different frequency components because of the restriction of the space-sampling law. Therefore, an array of multichannel geophones with small channel spacing is adopted in the existing multichannel transient Rayleigh wave detecting technique. With such kind of geophone array, a series of channel spacing ranging from the smallest spacing between a pair of adjacent geophones to the largest spacing between the two geophones at the two ends of the array, can be formed (Fig 11A). Through this method, the requirement of the channel spacing to simultaneously measure different frequency components is satisfied. By this method, however, only one (*V*_R_ − *Z*) curve can be acquired with such an array of multichannel geophones, which greatly limits the efficiency of detection.
2. The (*V*_R_ − *Z*) curve acquired via existing method can only reveal the average distribution of phase velocities on depths within the scope covered by the array along the detecting line, which is equivalent to performing only one velocity test in one borehole within the range covered by the geophone array (Fig 11A). Thus, the test results of the existing method of multichannel Rayleigh wave detecting technique cannot reveal the lateral variation of rock or soil masses within the range covered by the multichannel geophone array. In fact, due to the low lateral resolution of the existing multichannel transient Rayleigh wave detecting method, it is very difficult to apply this method to high precision, small scale detection.
3. The phase-difference method is used to extract the phase velocity of Rayleigh waves in the existing multichannel transient Rayleigh wave detection method. According to Eq (11), the phase difference ΔΦ_*k*_(*f*_*j*_) consists of two parts: ΔΦ_0*k*_(*f*_*j*_) and 2*πf*_*j*_*τ*_*k*_. Neglecting TDP, the existing multichannel transient Rayleigh wave detecting method cannot extract phase velocities corresponding to the high-frequency components of the Rayleigh wave precisely: the higher the frequency is, the greater the error will be, which will definitely influence the accuracy of the phase-velocity extraction. Consequently, the extracted phase velocities corresponding to the high-frequency components of the transient Rayleigh wave are quite unstable. Thus, the resolution of the transient Rayleigh wave detection for the high-frequency band is greatly limited because of the absence of TDP.

### Improvements of the proposed method by considering TDP

In this paper, several improvements to the multichannel transient Rayleigh wave detecting method are achieved by considering the concept TDP. First, the introduction of TDP enables the extraction of accurate phase differences of any frequency wave component with any channel spacing. As long as the rock or soil mass between two detection points is homogeneous, the channel spacing can be enlarged as much as possible because no error is induced in the phase difference by extracting the high-frequency components of the Rayleigh waves. Thus, the relative measurement accuracy of the time difference between two channels can be improved significantly. Second, the introduction of TDP also enables the extraction of phase differences of various frequency components from transient Rayleigh waveforms picked up by only one pair of geophones instead of a whole array of multichannel geophones; thus, from an array of *n* channel geophones, the improved method proposed in this paper can extract *n* − 1 curves of phase velocity vs. effective detecting depth (*V*_R_ − *Z*) from *n* − 1 pairs of adjacent detecting points on a detecting line with *n* detecting points (Fig 11B).

Consequently, the operating efficiency and lateral resolution of transient Rayleigh wave detecting are improved greatly. Moreover, the introduction of TDP eliminates the high-frequency threshold to phase difference calculation of transient Rayleigh waves and greatly improves the high-frequency resolution of the transient Rayleigh wave detecting method. After TDP is added to the analysis of the phase difference, in fact, the detection ability of the multichannel transient Rayleigh wave detecting method can be expanded to the ultrasonic frequency band. In addition, the method has achieved good results in the detection of weathering of white marble handrails in the Forbidden City [21].

### Further studies on the proposed method

By considering TDP in the multichannel transient Rayleigh wave detecting method, the limitation on the distance between channels in the arrangement of detecting points is eliminated. Therefore, the improved multichannel transient Rayleigh wave detecting method can be applied to detect multi-scale objects. In the development and construction of urban underground space, geophysical prospecting methods, such as the Rayleigh wave detecting method, can be applied first gain necessary information on the intricacies of the underground geology.

On the other hand, the three-dimensional imaging of the detecting results is also necessary for further application. For the present case study, Fig 10 shows quasi-3D imaging of underground karst water channels. Combined with a spatial data interpolation method and 3D geological modeling technique, this method can be applied to the analysis of spatial differences in rock and soil masses in detected areas and to propose the optimization of grouting position and grouting volume to seal karst water, which will greatly reduce the cost of the project and improve the reliability of construction.

Furthermore, in the proposed method, it is a key point to identify the seismic phase of Rayleigh wave in a recorded waveform (or wave train). As shown in Figs 4 and 6, a recorded waveform contains different seismic phases such as P wave, S wave and R wave. At present in the proposed method, the Rayleigh wave is distinguished from different seismic phases in the recorded waveform mainly by the first motion directions and relative amplitude variations of the seismic phases, and then the first-arrival time of the Rayleigh wave is picked up artificially. In fact, there is a certain relationship between wave amplitude and wave period [33, 34]. The amplitude-period relations of different seismic phases are different. Based on this point of view, it is necessary to carry out further studies on the amplitude-period relations of different seismic phases, which are of great significance to realize the automatic identification of Rayleigh wave and to improve the accuracy of its first-arrival time extracting, thus further to increase the efficiency and accuracy of the proposed method.

## Conclusions

To overcome the technical limitations of the existing transient Rayleigh wave detecting method, in this paper, the concept TDP (2*πfτ*) is defined, and the significance of the TDP in the extraction of phase velocities of Rayleigh waves is thoroughly discussed. On this basis, an improved multichannel transient Rayleigh wave detecting method considering TDP is proposed. The primary advancements made by the proposed method are as follows:

Firstly, the introduction of TDP enables the extraction of the correct phase difference of any frequency wave component with any channel spacing, which eliminates the limitation of space sampling law on wave detecting from the transient Rayleigh wave detecting method. This improvement enables the widening of channel spacing in the transient Rayleigh wave detecting method to the largest possible extent provided that the property of the medium between two detecting points is homogeneous. In this manner, the relative measurement accuracy of the time difference between two channels can be improved significantly.

Secondly, with only one pair of adjacent detection points, the phase differences of multiple frequency components of Rayleigh waves can be extracted. Consequently, *n* − 1 curves of phase velocity vs. effective detecting depth (*V*_R_ − *Z*) can be obtained from *n* − 1 pairs of adjacent detecting points on a detecting line with *n* detecting points. Thus, the operating efficiency and lateral resolution of the transient Rayleigh wave detecting method are greatly improved.

Third, the introduction of TDP eliminates the high-frequency threshold of the Rayleigh wave phase difference calculation, which is limited by the spacing distance between two adjacent detection points in existing methods; therefore, it has greatly improved the high-frequency resolution of the transient Rayleigh wave detecting method. Accordingly, the application area of the transient Rayleigh wave detecting method will be greatly enlarged.

The improved multichannel transient Rayleigh wave detection method was applied to the detection of karst water channels in the rock mass in the foundation pit of Yan’an Road Station of Guiyang Metro. Quasi-3D imaging intuitively revealed the scale and spatial distribution of underground karst water channels, thus providing strong technical support for treating karst water gushing problems in the excavation process of the station foundation pit. Following this, water gushing in the foundation pit could be successfully plugged.

## Supporting information

S1 Dataset Relevant data of this study. The relevant data of this study can be found from the Supporting Information file labeled “S1_Dataset”.
(RAR)
